# Alpha-Mangostin as a New Therapeutic Candidate for Concanavalin A-Induced Autoimmune Hepatitis: Impact on the SIRT1/Nrf2 and NF-κB Crosstalk

**DOI:** 10.3390/plants11182441

**Published:** 2022-09-19

**Authors:** Ahmed M. Shehata, Hossein M. Elbadawy, Sabrin R. M. Ibrahim, Gamal A. Mohamed, Wael M. Elsaed, Aisha A. Alhaddad, Nishat Ahmed, Hany Abo-Haded, Dina S. El-Agamy

**Affiliations:** 1 Department of Pharmacology and Toxicology, College of Pharmacy, Taibah University, Al-Madinah Al-Munawwarah 30078, Saudi Arabia; 2 Department of Pharmacology and Toxicology, Faculty of Pharmacy, Beni-Suef University, Beni-Suef 62511, Egypt; 3 Preparatory Year Program, Department of Chemistry, Batterjee Medical College, Jeddah 21442, Saudi Arabia; 4 Department of Pharmacognosy, Faculty of Pharmacy, Assiut University, Assiut 71526, Egypt; 5 Department of Natural Products and Alternative Medicine, Faculty of Pharmacy, King Abdulaziz University, Jeddah 21589, Saudi Arabia; 6 Department of Anatomy and Embryology, Faculty of Medicine, Mansoura University, Mansoura 35516, Egypt; 7 College of Medicine, Taibah University, Al-Madinah Al-Munawwarah 30078, Saudi Arabia; 8 Faculty of Medicine, Mansoura University, Mansoura 35516, Egypt; 9 Department of Pharmacology and Toxicology, Faculty of Pharmacy, Mansoura University, Mansoura 35516, Egypt

**Keywords:** *Garcinia mangostana*, α-mangostin, xanthones, concanavalin A, autoimmune hepatitis, SIRT1/Nrf2/NF-κB signaling

## Abstract

Alpha-mangostin (α-MN) is a xanthone obtained from *Garcinia mangostana* that has diverse anti-oxidative and anti-inflammatory potentials. However, its pharmacological activity against autoimmune hepatitis (AIH) has not been investigated before. Concanavalin A (Con A) was injected into mice to induce AIH and two doses of α-MN were tested for their protective effects against Con A-induced AIH. The results demonstrated the potent hepatoprotective activity of α-MN evidenced by a remarkable decrease of serum indices of the hepatic injury and amendment of the histological lesions. α-MN significantly attenuated the level and immuno-expression of myeloperoxidase (MPO) indicating a decrease in the neutrophil infiltration into the liver. Additionally, the recruitment of the CD4+ T cell was suppressed in the α-MN pre-treated animals. α-MN showed a potent ability to repress the Con A-induced oxidative stress evident by the reduced levels of malondialdehyde (MDA), 4-hydroxynonenal (4-HNE), and protein carbonyl (PC), as well as the enhanced levels of antioxidants as the reduced glutathione (GSH), superoxide dismutase (SOD), and total antioxidant capacity (TAC). The ELISA, RT-PCR, and IHC analyses revealed that α-MN enhanced the sirtuin1/nuclear factor erythroid 2 related factor-2 (SIRT1/Nrf2) signaling and its downstream cascade genes concurrently with the inhibition of the nuclear factor kappa B (NF-κB) and the inflammatory cytokines (tumor necrosis factor-alpha and interleukine-6) signaling. Taken together, these results inferred that the hepatoprotective activity of α-MN could prevent Con A-induced AIH through the modulation of the SIRT1/Nrf2/NF-κB signaling. Hence, α-MN may be considered as a promising candidate for AIH therapy.

## 1. Introduction

AIH (Autoimmune hepatitis) is a deleterious hepatic inflammation with an indistinct etiology and an exacerbation in the mortality rate if not treated. This condition can end up as cirrhosis, and nearly 1% of AIH patients may experience significant worsening, such as the fulminant hepatic failure. The inflammatory cells infiltrating into the hepatic tissue are the substantiating characteristics of AIH in addition to the augmented levels of aminotransferases, IgG, and autoantibodies. The approach to AIH is a clinical challenge as it is a polygenic disease in which the severity and complexity vary among patients, from hepatocellular necrosis to liver cirrhosis, that necessitates a liver transplant [1,2]. The pathogenesis of AIH is complex and involve multiple pathophysiological events. Among many factors, the excessive generation of free radicals and subsequent lipid peroxidation/oxidative damage are important factors that potentiate the development of AIH [3,4]. The inflammatory responses in AIH are greatly mediated by the release of cytokines as TNF-α (tumor necrosis factor), interleukins (ILs), and interferon (IFN)-γ, which are regulated by the interplay of many signaling pathways especially NF-κB (nuclear factor-kappa B) and the nuclear factor (erythroid-derived 2)-like 2 (Nrf2) signaling. NF-κB is an important transcriptional modulator of cellular inflammatory responses via the induction of the pro-inflammatory cytokines’ expression. Nrf2, a redox-sensitive transcription factor, controls the cellular antioxidant defenses via the induction of the antioxidant genes expressions. The crosstalk between Nrf2 and NF-κB plays a pivotal role in AIH and recently has become a hot spot for many studies [5,6,7].

A convenient animal model that resembles AIH in humans has been well established using a single dose of concanavalin A (Con A) which is a lectin derived from the *Canavalia ensiformis* extract [8]. Con A-induced hepatitis has a ubiquitous role and specificity in the activation of direct T-cells. It has similarities in autoimmune models with humans, drug-induced hepatotoxicity which is immune-mediated, and viral hepatitis [9]. A Con A administration induces the activation of CD4+ T helper cells which play a pivotal role in the immune system regulation and modulating the release of inflammatory mediators such as TNF-α and ILs, which promote the hepatic damage [6]. Hence, Con A-induced AIH is widely used to test the efficacy of new hepatoprotective candidates that could be used for AIH.

**Figure 1:**
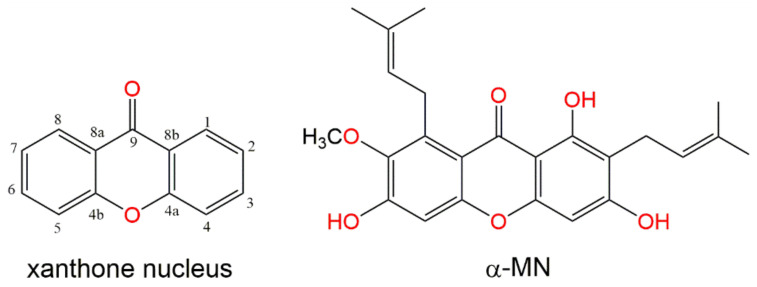
Xanthone nucleus and the α-MN structure.

Fruits are highly consumed and largely appreciated throughout the world. *Garcinia* is the biggest genus of Clusiaceae family, that includes ≈400 species [10]. *Garcinia mangostana* is a round, reddish, or dark purple fruit, which has a slightly acidic and sweet flavor and high nutritional value. It has been traditionally used to treat a great variety of medical conditions. *G. mangostana* is rich in xanthones which are tricyclic (C6-C3-C6), oxygenated compounds that have a dihydrofuran ring, methoxy, isoprene, and hydroxyl groups at different positions on the xanthene-9-one skeleton (Figure 1), resulting in a diverse array of derivatives. *G. mangostana* xanthones revealed various bioactivities: α-amylase inhibitory, anti-leishmanial, anti-HIV, antimicrobial, antioxidant, antihypertensive, antiquorum sensing, anti-inflammatory, antimalarial, and cytotoxic [11,12,13,14,15,16]. Alpha-mangostin (α-MN) is one of the major *G. mangostana* xanthones that possesses diverse bioactivities, such as anti-tumor, anti-inflammatory, cardio-protective, antibacterial, anti-diabetic, larvicidal, antifungal, α-amylase inhibitory, antiallergic, anti-parasitic, anti-obesity, and antioxidant [17,18,19]. Moreover, it is applied in cosmetics as an anti-wrinkle and anti-aging ingredient, as well as for the treatment of acne and for maintaining skin lubrication [18]. Many studies have revealed the therapeutic potential of α-MN regarding liver-related disorders. α-MN was found to decrease thioacetamide-induced liver cirrhosis in rats [20]. It attenuated the hepatic steatosis through the improvement of mitochondrial functions and enhancing the cellular antioxidant capacity in high fat-diet fed rats [21]. Further, it prohibited the hepatic stellate cells activation and proliferation induced by acetaldehyde through the inhibition of TGF-β and ERK 1/2 pathways in obese mice [22]. However, its potential on AIH has not been assessed yet. Therefore, this study is targeted to evaluate the hepatoprotective efficacy of α-MN isolated from *G. mangostana* against Con A-induced AIH and to investigate its impact on the oxidative/inflammatory molecular signaling pathways.

## 2. Materials and Methods

### 2.1. General Procedures

*ESIMS* was acquired using a Mass LCQ DECA spectrometer (ThermoFinnigan, Bremen, Germany). The NMR data were recorded on a 600 MHz Bruker spectrometer (Bruker BioSpin, Billerica, MA, USA). The chromatographic separation and the TLC analysis were accomplished using SiO_2_ 60 and SiO_2_ 60 F_254_ plates, respectively. The HRESIMS (high resolution electron spray ionization mass spectroscopy) was measured using a LTQ Orbitrap mass spectrometer (Thermo-Scientific, Bremen, Germany).

### 2.2. Extraction and Isolation of α-MN

*G. mangostana* fruits were procured from a Saudi local market. Its verification was accomplished as formerly stated and a voucher (no. GM1424) specimen was kept in the Faculty of Pharmacy’s herbarium [23]. The dried pericarps (1.0 kg) were extracted with MeOH (5 L × 5) until exhaustion at room temperature and the combined extracts were evaporated under vacuum. The MeOH extract (GMT, 120 g) was suspended in distilled H_2_O (200 mL) and partitioned between EtOAc and *n*-hexane to produce *n*-hexane (7.9 g), EtOAc (40.3 g), and aqueous (67.8 g) fractions. The SiO_2_ CC of EtOAc fraction using the *n*-hexane/EtOAc gradient afforded four subfractions: GME-1–GME-4. The GME-2 (8.1 g) subfraction was separated on SiO_2_ CC using the EtOAc/*n*-hexane gradient to get α-MN (3.7 g).

#### Spectral Data of α-MN

Yellow amorphous powder; ^1^H NMR (CDCl_3_/600 MHz): δ_H_ 13.77 (1OH), 6.82 (H5), 6.29 (H4), 5.28 (H2’), 5.25 (H2’’), 4.08 (H1’’), 3.80 (7OCH_3_), 3.45 (H1’), 1.84 (H5’’), 1.83 (H5’), 1.76 (H4’’), 1.69 (H4’); ^13^C NMR (CDCl_3_/125 MHz): δ_H_ 182.1 (C9), 161.7 (C3), 161.6 (C1), 155.8 (C6), 155.1 (C4a), 154.6 (C4b), 142.6 (C7), 137.1 (C8), 135.8 (C3’), 132.2 (C3’’), 123.2 (C2’’), 121.5 (C2’), 112.2 (C8a), 108.5 (C2), 103.6 (C8b), 101.6 (C5), 93.3 (C4), 62.1 (7OCH_3_), 26.6 (C1’’), 25.9 (C4’’), 25.8 (C4’), 21.5 (C1’), 18.3 (C5’’), 17.9 (C5’); ESIMS *m*/*z*: 411 [M+H]^+^; HRESIMS *m*/*z*: 411.1803 [M+H]^+^ (calcd for C_24_H_27_O_6_, 411.1808) [15,24].

### 2.3. Animals

Adult male Swiss albino mice (20–25 g) were held under standard laboratory conditions adjusted at 25 °C, 60% humidity, and an alternate 12 h light/shade cycle. The animals had access freely to water and standard rodent food. The experimental procedures and protocol were authorized by the Research Ethics Committee of the College of Pharmacy, Taibah University, Saudi Arabia (reference number: COPTU-REC-19-20210524) which sticks to “Principles of Laboratory Animals Care” (NIH publication).

### 2.4. Induction of AIH and Experimental Groups

The Con A was dissolved in normal saline (Sigma-Aldrich, St. Louis, MO, USA) and was used to induce AIH via one intravenous dose (15 mg/kg) as described previously [8,25]. The mice were assigned to five groups (eight mice each) and received the treatment as follows:

1. Control: the vehicle for 7 days.
2. α-MN: only α-MN (50 mg/kg, dissolved in soybean oil) orally for 7 days.
3. Con A: only Con A (15 mg/kg, single IV dose).
4. α-MN 25 + Con A, mice were given α-MN (25 mg/kg, orally once a day for 7 days) prior to the Con A injection in the last day.
5. α-MN 50 + Con A, mice were given α-MN (50 mg/kg, orally once a day for 7 days) prior to the Con A injection in the last day.

Twelve hours after the Con A challenge, the mice were humanly killed using an overdose of anesthesia with diethyl ether. The blood was collected from all mice and then centrifuged to obtain the serum that was kept at −20 °C until analyzed. The liver from each mouse was collected and samples were obtained for subsequent diverse estimations. The preparation of the hepatic homogenates was carried out using a phosphate buffered saline (PBS). Briefly, a small piece of the hepatic tissue was homogenized in the PBS (about 10 mg tissue to 100 µL PBS). Then it was centrifuged for 20 min at 1500× *g* (or 5000 rpm) in order to obtain the supernatants that were kept at −80 °C for further analysis. Other hepatic samples were flesh frozen in liquid nitrogen and kept at −80 °C until used for the RNA extraction for the PCR measurements. Other liver samples were fixed in buffered formalin and embedded in paraffin to be used for the histopathological and immunohistochemical examinations.

### 2.5. Biochemical Investigation

#### 2.5.1. Serum Indices of AIH

Aspartate aminotransferase (AST), alanine aminotransferase (ALT), alkaline phosphatase (ALP), lactate dehydrogenase (LDH), γ-glutamyl transferase (γ-GT), and albumin were estimated in the serum using available commercial assay kits (Human, Wiesbaden, Germany; Spectrum Co., Cairo, Egypt).

#### 2.5.2. Myeloperoxidase (MPO)

MPO was measured in the hepatic supernatants as an index for neutrophil infiltration using a commercially available kit (Abcam, Cambridge, UK).

#### 2.5.3. Oxidative Parameters and Antioxidants

Indicators of lipid peroxidation (e.g., malondialdehyde (MDA), protein carbonyl (PC), and 4-hydroxyenonenal (4-HNE)) and antioxidants (e.g., TAC (total antioxidant capacity), GSH (reduced glutathione), and SOD (superoxide dismutase)) were assessed in the hepatic homogenates’ supernatants utilizing the available kits (MyBioSource Inc., San Diego, CA, USA; Bio-Diagnostic, Giza, Egypt).

#### 2.5.4. NF-κB and Inflammatory Cytokines

NF-κB, IL-6, and TNF-α were analyzed in the supernatants of the liver homogenates using ELISA kits (CUSABIO, Wuhan, China; R&D Systems Inc., Minneapolis, MN, USA).

#### 2.5.5. Nrf2 and Heme Oxygenase-1 (HO-1)

The Nrf2 binding activity was estimated in the nuclear extracts (about 5 μg of nuclear protein) based on the instructions of the kit (TransAM Nrf2 kit, Active Motif Inc., Carlsbad, CA, USA). Briefly, on ice, a small piece of fresh hepatic tissue was mixed with an ice-cold hypotonic extraction buffer containing dithiothreitol, detergent, phosphatase, and protease inhibitors. The mixture was incubated for 15 min and then centrifuged for 10 min at 4 °C. The cell pellets were resuspended in the hypotonic extraction buffer and incubated for 15 min on ice with vortex every 3 min. The supernatants were obtained after the centrifugation at 4 °C and their protein content was estimated. HO-1 was estimated in the liver homogenates supernatants using an ELISA kit (MyBioSource Inc., San Diego, CA, USA).

### 2.6. Liver Histopathology

The specimens were stained with haematoxylin and eosin. The total surface of the whole slides was examined and scored. The degree of lesion was estimated and graded (Grade 0–4) as previously described, based on the severity of necrosis and inflammation: none: 0; very mild: 1; mild (≤30%): 2; moderate (≤60%): 3; and severe (≥60%): 4 [25]. Whole sections of a minimum of 10 slides per animal were examined using an optical microscope (Olympus, Tokyo, Japan). The images were visualized, photographed, and analyzed using the ImageJ software (NIH, Bethesda, MD, USA).

### 2.7. RT-PCR Assessment

The genetic expression of SIRT1, Nrf2, HO-1, GCLc, NQO1, NF-κBp65, HO-1, IL-6, and TNF-α was estimated as described above [6]. In brief, the total RNA was extracted from each hepatic sample (≈30 mg) using a RNeasy MiniKit following the provided instructions (Qiagen, Germantown, MD, USA). The ratio A260/A280 was used to check the purity of the RNA and only the pure RNA (ratio of 1.8–2.1) samples were selected and used. Complementary DNA (cDNA) was obtained from the RNA using a cDNA reverse transcription kit (Qiagen, Germantown, MD, USA). Finally, the RT-PCR was carried out using SYBR Green PCR Master Mix on the thermocycler-Rotor-Gene Q. The primers sequences for the targeted genes are shown in Table 1. The target mRNAs relative levels were normalized to β-actin and calculated using the ^ΔΔ^Ct method.

### 2.8. Immunohistochemistry (IHC)

The automatic IHC estimation was performed using the Ventana-Bench-Mark-XT system according to the formerly described procedures [8]. Briefly, the paraffin-sections of the hepatic tissue were dewaxed using xylene and graded alcohols and then rehydrated before boiling in a citrate buffer (10 mM, pH 6) for 10 min and cooling at 25 °C for 20 min. The endogenous peroxidase activity was blocked by immersing the slides in a buffer containing hydrogen peroxide (3%). The immuno-staining was performed using the primary antibodies: rabbit polyclonal antibody against MPO (1:150, Fisher Scientific Inc. Waltham, MA, USA), CD4+ (1:100, Abnova, Taipie, Taiwan), SIRT1 (1:200, Fisher Scientific Inc., Waltham, MA, USA), NF-ĸB p65 (1:200, Fisher Scientific Inc., Waltham, MA, USA), TNF-α (1:100, Fisher Scientific Inc., Waltham, MA, USA), and IL-6 (1:100, Abcam, Cambridge, UK).The visualization was carried out using diaminobenzidine (DAB) and counterstain using hematoxylin. The slide analysis (10 slides/each animal) was achieved using the image analysis software (ImageJ, NIH).

### 2.9. Statistics

The results were presented as mean ± S.E. A One-way analysis of variance (ANOVA) followed by Tukey’s Kramer-multiple comparison test were adapted for the comparison of various groups. The GraphPad Prism software (v.8.01; GraphPad Software, La Jolla, Cam, San Diego, CA, USA) was used for the analysis. The statistical significance was identified at *p* < 0.05.

## 3. Results

**Figure 2:**
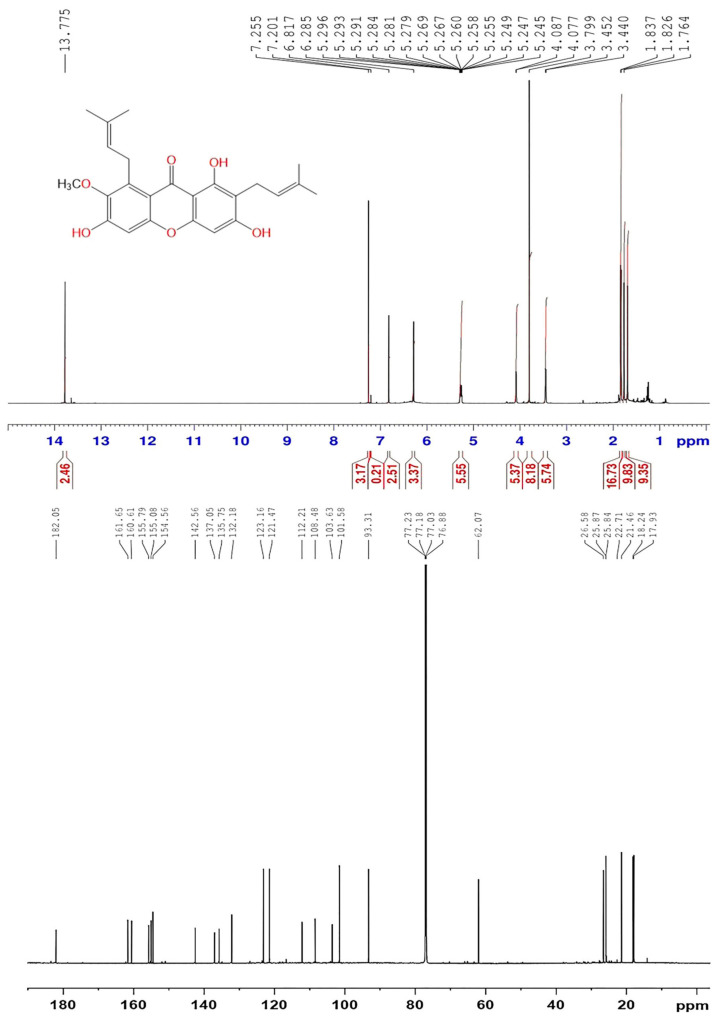
^1^H (600 MHz) and ^13^C (150 MHz) NMR spectra of α-MN in CDCl_3_.

The GM extract was subjected to repeated SiO_2_ CC using *n*-hexane/EtOAc gradient to yield α-MN. Its structure was verified by the comparison of the spectroscopic (NMR and MS) (Figure 2) and physicochemical data with the literature [15,24].

### 3.1. α-MN Alleviated Con A-Induced Hepatic Lesions

**Figure 3:**
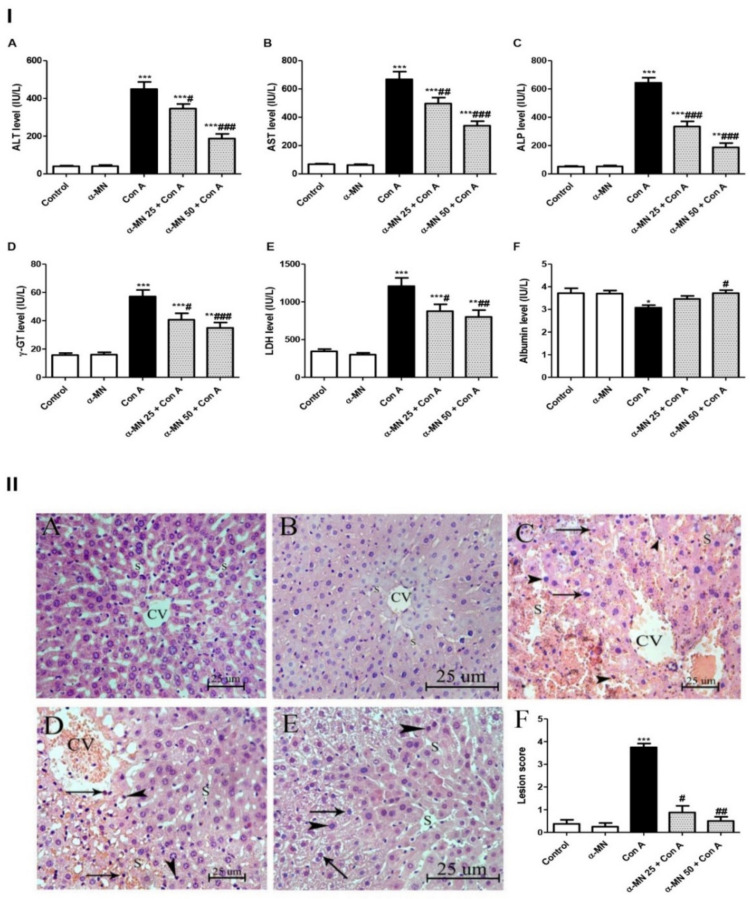
Alpha-mangostin (α-MN) conferred protection against concanavalin-A (Con A)-induced hepatitis: improvement of biochemical and histopathological indices of the hepatic injury. (**I**) (**A**): Alanine aminotransferase (ALT); (**B**): Aspartate aminotransferase (AST); (**C**): Alkaline phosphatase (ALP); (**D**): Gamma-glutamyl transferase (γ-GT); (**E**): Lactate dehydrogenase (LDH); (**F**): Albumin. (**II**) Hepatic sections stained with H&E (×400); (**A**): Control and (**B**): α-MN groups displayed normal hepatic architecture with the hepatocytes arranged in regular plates separated by the blood sinusoids (S) and radiating from around the CV (central vein); (**C**): Con A group showed marked hepatocytes trabeculation, hydropic degeneration, and apoptotic changes (arrow heads), diffuse intense sinusoidal congestion (S) and several inflammatory cell infiltrations (arrows); (**D**): α-MN 25 + Con A group showed alternating areas of multiacinar necrosis with the same pathological changes with areas of a normal hepatic structure; (**E**): α-MN 50 + Con A group showed a marked improvement with still hepatocytes having a vacculated cytoplasm and a less marked apoptotic nuclei and inflammatory cell infiltration; (**F**): Scores of the histopathological hepatic lesion. Data are the mean ± SE. (*n* = 8); *** *p* < 0.001; ** *p* < 0.01; * *p* < 0.05 vs. control group; ^###^ *p* < 0.001; ^##^ *p* < 0.01, ^#^ *p* < 0.05 vs. Con A group (one-way ANOVA).

The Con A injection induced notable hepatic damage that was evident through the significant rise in the serum indices of the liver toxicity (ALT, ALP, LDH, AST, and γ-GT) and significant depression in the albumin level compared with the control group (Figure 3I).

The biochemical analysis was in the same line with the histopathological results. The hepatic specimen of the Con A group exhibited delirious hepatic injury. Inflammatory changes such as several inflammatory cell infiltrations and hydropic degeneration, as well as apoptotic changes were observed in the Con A group while the control group displayed a normal hepatic architecture (Figure 3II).

Interestingly, the α-MN pretreated groups exhibited remarkable improvement in the hepatic inflammatory state. The α-MN pretreatment ameliorated the elevated hepatotoxicity indices and increased the albumin level compared with the Con A group. Furthermore, α-MN improved the liver histopathology and ameliorated the hepatic lesions.

### 3.2. α-MN Decreased the Con A-Induced Infiltration of the Neutrophiles and the CD4+ T Cells into the Liver

**Figure 4:**
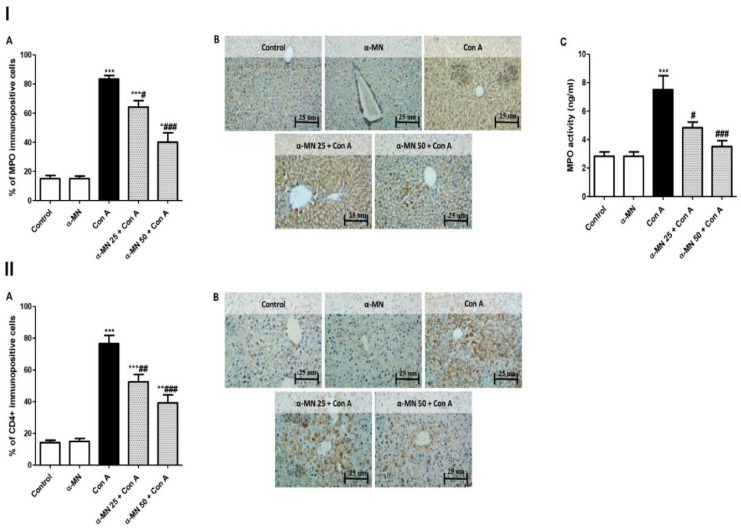
Alpha-mangostin (α-MN) decreased concanavalin A (Con A)-induced infiltration of the neutrophiles and the CD4+ T cells into the liver. (**I**) MPO immuno-expression and level; (**II**) Immuno-expression of CD4+ in the hepatic specimen. The immuno-staining of MPO and CD4+ of the control and α-MN groups was minimal while that of Con A was high; α-MN pre-treated groups showed a remarkable lowered level of immunostaining for both MPO and CD4+ (×200). Data are the mean ± SE. (*n* = 8); *** *p* < 0.001; ** *p* < 0.01; * *p* < 0.05 vs. control group; ^###^ *p* < 0.001; ^##^ *p* < 0.01, ^#^ *p* < 0.05 vs. Con A group (one-way ANOVA).

The Con A group exhibited an elevated level and a high MPO immuno-expression compared with normal group (Figure 4I). On the contrary, the α-MN pre-treatment dramatically decreased the MPO level and its immuno-expression compared with the Con A group. Additionally, a Con A injection augmented the CD4+ T cells infiltration into the hepatic tissue compared with the control mice. The α-MN pre-treatment suppressed the CD4+ T cells infiltration in comparison with the Con A group (Figure 4II).

### 3.3. α-MN Ameliorated the Con A-Induced Oxidative Stress and the Enhanced Antioxidants

**Figure 5:**
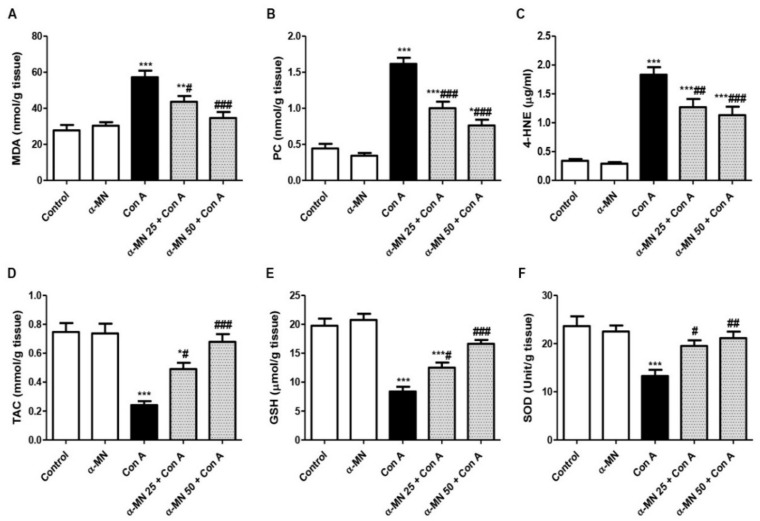
Alpha-mangostin (α-MN) ameliorated concanavalin-A (Con A)-induced oxidative stress and enhanced antioxidants in the hepatic tissue. (**A**) Malondialdehyde (MDA); (**B**) Protein carbonyl (PC); (**C**) 4-Hydroxynonenal (4-HNE); (**D**) Total antioxidant capacity (TAC); (**E**) Reduced glutathione (GSH); (**F**) Superoxide dismutase (SOD). Data are the mean ± SE. (*n* = 8); *** *p* < 0.001; ** *p* < 0.01; * *p* < 0.05 vs. control group; ^###^ *p* < 0.001; ^##^ *p* < 0.01, ^#^ *p* < 0.05 vs. Con A group (one-way ANOVA).

As demonstrated in Figure 5, the Con A injection resulted in a significant increase in the oxidative parameters (MDA, PC, and 4-HNE) compared with the normal mice that was concurrent with the depression in the hepatic antioxidants (TAC, GSH, and SOD). Moreover, α-MN succeeded to ameliorate the oxidative burden as it significantly decreased the lipid peroxidative products and boosted the antioxidants in comparison with the Con A group.

### 3.4. α-MN Counteracted the Con A-Induced Suppression in the SIRT1/Nrf2/HO-1 Signaling

**Figure 6:**
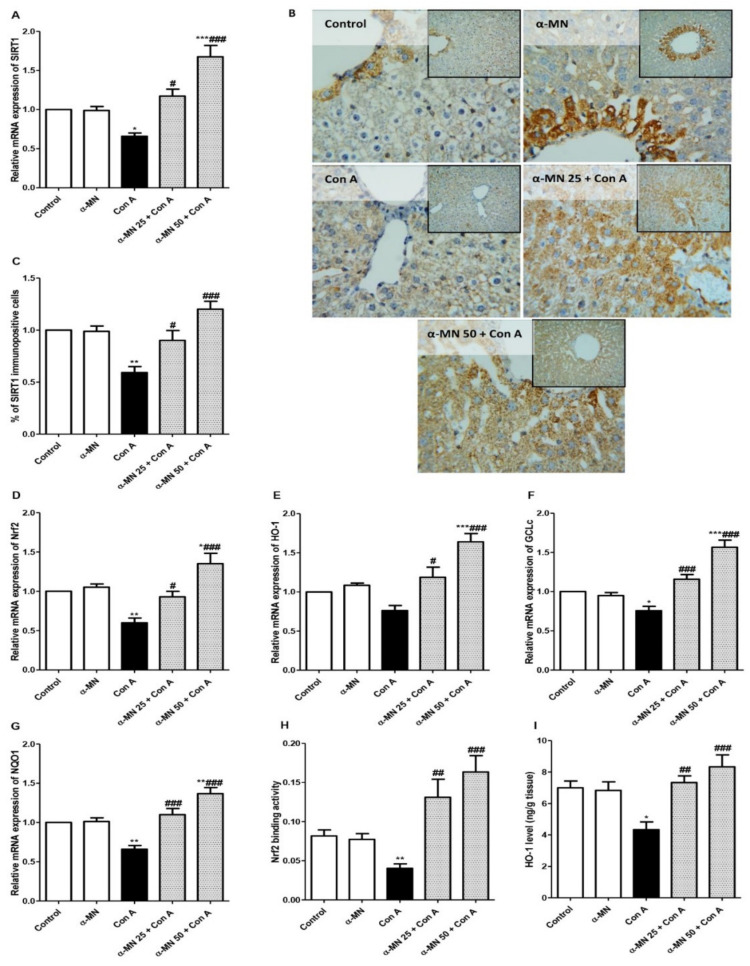
Alpha-mangostin (α-MN) counteracted concanavalin-A (Con A)-induced suppression in the SIRT1/Nrf2/HO-1 signaling. (**A**–**C**) mRNA and protein expression of SIRT1 showing an enhanced protein immuno-expression of SIRT1 in the α-MN pretreated groups; (**D**–**G**) mRNA and protein expression of Nrf2, HO-1, GClc, GCLm, NQO1; (**H**) Nrf2 binding activity; (**I**) HO-1 level. Data are the mean ± SE. (*n* = 8); *** *p* < 0.001; ** *p* < 0.01; * *p* < 0.05 vs. control group; ^###^ *p* < 0.001; ^##^ *p* < 0.01, ^#^ *p* < 0.05 vs. Con A group (one-way ANOVA).

The Con A challenge resulted in a reduced mRNA expression of SIRT1, Nrf2, NQO1, and GCL in comparison with the control group. The immuno-expression of SIRT1 was also depressed in the Con A group compared with normal animals. The Nrf2 binding capacity and HO-1 level were significantly decreased in the Con A group. On the contrary, the α-MN pre-treatment amplified the Nrf2/SIRT1/NQO1/HO-1/GCL signaling and augmented the expression of these genes. Moreover, the Nrf2 binding capacity and HO-1 level were increased in the α-MN pre-treated groups compared with the Con A group (Figure 6).

### 3.5. α-MN Prohibited the NF-κB/TNF-α/IL-6 Signaling Activation Induced by Con A

**Figure 7:**
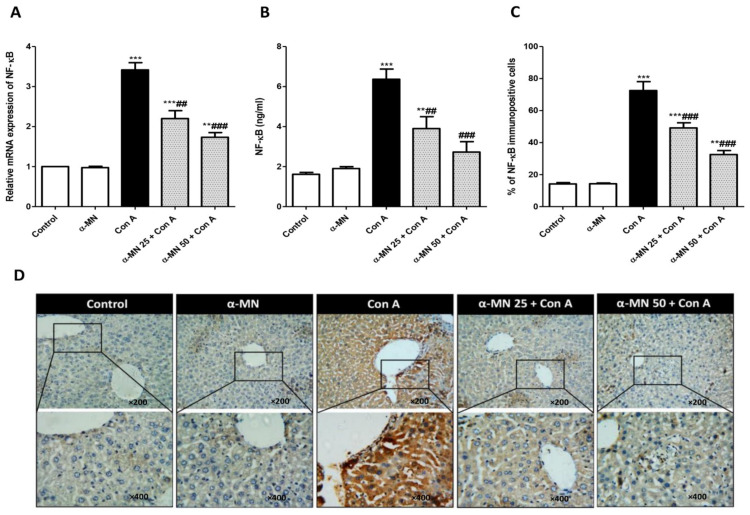
Alpha-mangostin (α-MN) inhibited concanavalin-A (Con A)-induced activation of the NF-ĸB signaling in the hepatic tissue. (**A**) mRNA expression NF-ĸB p65; (**B**) Level of NF-ĸB; (**C**) % of the NF-ĸB immuno-positive cells; (**D**) Immuno-expression of NF-ĸB where control and α-MN groups exhibited minimal NF-ĸB immuno-staining, Con A group showed intense NF-ĸB immuno-staining; Both groups of α-MN + Con A exhibited low immuno-staining for NF-ĸB. Data are the mean ± SE. (*n* = 8). ** *p* < 0.01, *** *p* < 0.001 vs. control group; ^##^ *p* < 0.01, ^###^ *p* < 0.001 vs. Con A group (one-way ANOVA).

**Figure 8:**
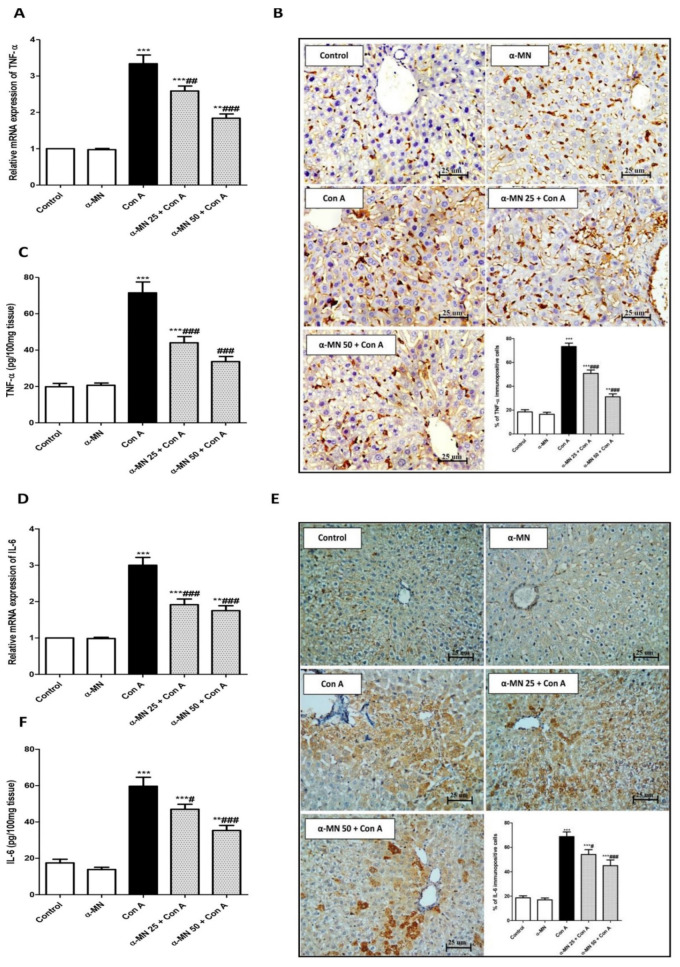
Alpha-mangostin (α-MN) decreased concanavalin-A (Con A)-induced enhancement of the TNF-α/IL-6 signaling in the hepatic tissue. (**A**) mRNA expression of TNF-α; (**B**) Immuno-expression of TNF-α (×400); (**C**) Level of TNF-α; (**D**) mRNA expression of IL-6; (**E**) Immuno-expression of IL-6 (×400); (**F**) Level of IL-6. The immuno-expression of TNF-α and IL-6 was minimal in α-MN pretreated groups compared to intense staining in the Con A group. Data are the mean ± SE. (*n* = 8). ** *p* < 0.01, *** *p* < 0.001 vs. control group; ^#^ *p* < 0.05, ^##^ *p* < 0.01, ^###^ *p* < 0.001 vs. Con A group (one-way ANOVA).

As shown in Figure 7, there was a significant increase in the mRNA expression, the level and immuno-expression of IL-6, NF-κB, and TNF-α in the Con A group in comparison with the control mice. Contrarily, the α-MN pre-treated groups showed significantly decreased NF-κB, TNF-α, and IL-6 levels and expression compared with the Con A group (Figure 7 and Figure 8).

## 4. Discussion

α-MN has various pharmacological potentials including antioxidant and anti-inflammatory activities. It has a protective potential on oxidative stress-mediated damage in various organs such as the heart, kidneys, and brain tissues [26,27,28]. The results of the present study demonstrated a new therapeutic potential for α-MN as it exhibited beneficial effects on Con A-induced AIH shown by the marked reduction in the serum indices of the hepatic lesions and the attenuation in the hepatic histopathological injuries in a dose-dependent way. α-MN significantly lessened the infiltration of the inflammatory cells, CD4+ T cells into the liver. Furthermore, the α-MN pre-treatment remarkably diminished the oxidative stress and inflammatory response induced by Con A. The hepatoprotective effects of α-MN against Con A-induced AIH may be ascribed to the modulation of the SIRT1/NRF2/NF-κB signaling pathways.

The rodent experimental model of Con A-induced AIH closely resembles the human viral and autoimmune hepatitis. A single Con A injection results in a deleterious AIH within 8–12 h. The elevated serum aminotransferase is an important index of hepatitis. These cytosolic enzymes are released into the blood stream after the hepatocyte death [6,29]. Our biochemical results confirmed the development of AIH after the Con A injection as evidenced by the elevated aminotransferases, ALP, and LDH. In addition, the albumin level was decreased pointing to a marked hepatic dysfunction. In the same line, the histopathological analysis displayed severe inflammatory hepatic lesions in the Con A group. Additionally, the Con A-caused neutrophil infiltration was confirmed by the elevated MPO level and the immuno-expression in the hepatic tissue. Furthermore, our results showed the recruitment of CD4+ T cells into the liver following the Con A injection, which play a pivotal role in mediating the inflammatory response in Con A-induced AIH. The results agreed with the previous studies that demonstrated the development of a deleterious AIH after a single injection of Con A [1,4,6]. On the contrary, α-MN significantly attenuated these biochemical indices of the hepatic injury and the histopathological lesions had almost completely disappeared in the α-MN pre-treated groups. Furthermore, the neutrophil infiltration and the CD4+ T cells recruitment into the liver were attenuated. Hence, these results demonstrated the ability of α-MN to preserve the integrity and function of the hepatic tissue, suggesting its potent protective effects on Con A-induced AIH. These findings added a new aspect to the hepatoprotective activity of α-MN which was discussed formerly against other models of induced hepatotoxicity as against hepatic steatosis [30], lipopolysaccharide/D-galactosamine-induced acute liver failure, acetaminophen-produced acute liver injury [31,32] and hepatocellular carcinoma [33].

The pathogenic events that underly Con A-induced AIH are numerous and include oxidative stress, SIRT1, Nrf2, and NF-κB signaling [34]. Following the Con A challenge, the redox imbalance due to the excessive generation and accumulation of the reactive oxygen species increases the cellular oxidative burden, which results in DNA damage [3], mitochondrial dysfunction, and exacerbation of inflammatory reactions leading to hepatocellular necrosis and apoptosis [7,29]. Our results revealed that the level of oxidative markers (MDA, 4-HNE, and PC) was remarkably increased, concurrent with the noticeable depression in the level of antioxidants (SOD, TAC, and GSH) in the liver of the Con A group, thus indicating that the lipid peroxidation was pronounced with the Con A administration. These results emphasized the data from previous studies [4,8,35]. Reversely, the α-MN pre-treatment reduced the level of peroxidative markers and augmented the antioxidants in the hepatic tissue indicating the α-MN ability to counteract the oxidative stress, which may contribute to its hepatoprotective potential. These data are in harmony with previous studies that demonstrated the antioxidant capacity of α-MN against experimental oxidative injuries [31,32,36,37,38].

The correlation between oxidative stress and cellular molecular defense mechanisms such as SIRT1/Nrf2 has been a hot spot for many studies. SIRT1 (NAD+-dependent histone deacetylase) can induce the antioxidant defense pathways and hence, represses the oxidative inflammatory lesions. Previous studies had pointed to the role of the SIRT1 upregulation in protecting against Con A-induced AIH [34,39,40]. Recent studies have focused on the link between the SIRT1 enhancement and subsequent activation of the Nrf2/inhibition of the NF-κB p65 signaling [41,42]. In a normal state, Nrf2 is captured in cytoplasm by Keap-1. The oxidative stress condition activates the Nrf2 signaling causing its dissociation from Keap-1, the translocation to the nucleus, and induction of the expression of the antioxidant cytoprotective genes (e.g., *NQO-1*, *GCL*, and *HO-1*) leading to the protection against oxidative injury [5,43]. The Con A challenge is associated with the inhibition of the Nrf2 signaling causing the downregulation of the antioxidant enzymes [7,25]. The current results confirmed the downregulation of Nrf2/SIRT1 and its downstream genes after the Con A administration. This was accompanied by the significant reduction of the Nrf2 binding potential and HO-1 level. Interestingly, these changes were repressed by the α-MN pre-treatment as there was an enhancement in the gene expression of Nrf2/ SIRT1/GCL/NQO1/HO-1 simultaneous with the augmented Nrf2 binding potential and HO-1 level. These results agree with the previous studies that showed the ability of α-MN to upregulate SIRT1/Nrf2 [31,32,44]. This indicated that the hepatoprotection of α-MN may be linked with its ability to enhance the SIRT1/Nrf2 signaling.

Another major modulator of the pathogenesis of Con A-induced AIH is the excessive generation of inflammatory cytokines. Notably, many reports have suggested that the inflammatory response may be the primary player in the mediation of a Con A-induced hepatitis [25,45]. Recent research demonstrated the increased level of TNF-α, ILs, and IFN-γ in Con A-induced hepatitis [4,5,34]. A TNF-α overproduction mitigates the release of other cytokines causing the generation of adhesive molecules and activation of the inflammatory cells. Moreover, TNF-α through its subsequent binding to receptors led to apoptosis and necrosis [45]. The cytokine release and expression are controlled by multiple pathogenic events including the activation of the NF-κB signaling [5,7,35]. Con A causes the activation of NF-κB leading to the expression of pro-inflammatory cytokines [4,46]. In agreement with previous findings, our data displayed that the Con A injection resulted in the NF-κB activation and the subsequent release and expression of TNF-α and IL-6. Expectedly, these events were reversed in the α-MN pre-treated groups. This indicated the inhibitory effect of α-MN on the activation of the NF-κB signaling, that is consistent with the previous research in which α-MN significantly prohibited the activation of NF-κB in LPS-induced microglial inflammatory responses [47], collagen-induced arthritis [48] and dextran sulfate sodium-induced colitis [49].

## 5. Conclusions

**Figure 9:**
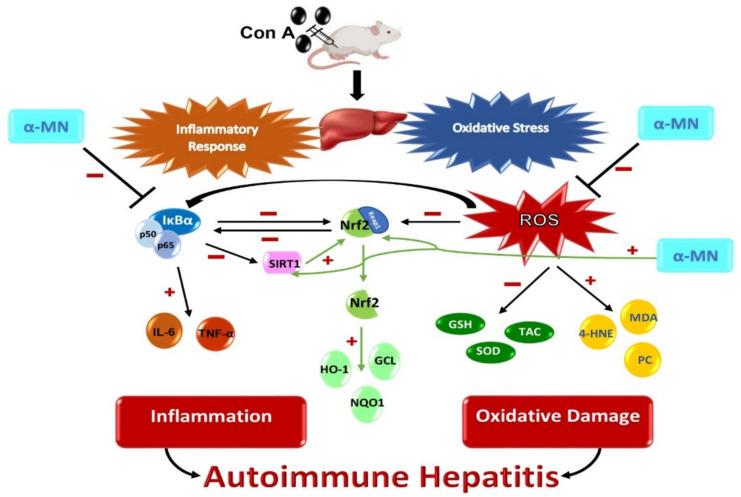
Schematic diagram showing the possible molecular mechanisms of the hepatoprotective potential of alpha-mangostin (α-MN) against concanavalin-A (Con A)-induced hepatitis.

α-MN could prevent Con A-induced AIH through its inhibitory effect on the hepatic oxidative stress and the inflammatory NF-κB/cytokine cascade, as well as the enhancement of the SIRT1/Nrf2 antioxidative signaling pathway (Figure 9). These results suggested a new scope for the use of α-MN in the treatment of AIH. Additional experimental work is mandatory to test the ability of α-MN to reverse the deleterious Con A-induced hepatic fibrosis and explore other mechanistic possibilities. Furthermore, clinical studies are essential to confirm the hepatoprotective efficacy of α-MN in AIH.

## Figures and Tables

**Table 1:** The sequence of the primers employed in RT-PCR.

Gene (Mouse)	Accession	PCR Product (bp)	Sequence (5′-3′)
*SIRT1*	NM_019812.3	111	**F:** CGATGACAGAACGTCACACG
			**R:** ATTGTTCGAGGATCGGTGCC
*Nrf2*	NM_010902	170	**F:** AAGAATAAAGTCGCCGCCCA
			**R:** AGATACAAGGTGCTGAGCCG
*HO-1*	NM_010442	200	**F:** CCTCACAGATGGCGTCACTT
			**R:** TGGGGGCCAGTATTGCATTT
*GCLc*	NM_010295	182	**F:** CTTTGGGTCGCAAGTAGGAAGC
			**R:** GGGCGTCCCGTCCGTTC
*NQO1*	NM_008706	111	**F:** CATTGCAGTGGTTTGGGGTG
			**R:** TCTGGAAAGGACCGTTGTCG
*NF-ĸB*	AY521463.1	102	**F:** AGGAAGGCAAAGCGAATCCA
			**R:** TCAGAACCAAGAAGGACGCC
*TNF-α*	NM_011609.4	102	**F:** GCTGTTGCCCCTGGTTATCT
			**R:** ATGGAGTAGACTTCGGGCCT
*IL-6*	NM_031168.2	79	**F:** AGTCCTTCCTACCCCAATTTCC
			**R:** GGTCTTGGTCCTTAGCCACT
*β-actin*	NM_007393.5	198	**F:** TGAGCTGCGTTTTACACCCT
			**R:** GCCTTCACCGTTCCAGTTTT

## Data Availability

Data are contained within the article.
